# Injury free Coalition for Kids® forges on amid the COVID-19 pandemic

**DOI:** 10.1186/s40621-021-00340-y

**Published:** 2021-09-13

**Authors:** Michael N. Levas

**Affiliations:** 1 Department of Pediatrics, Section of Emergency Medicine, Medical College of Wisconsin, Milwaukee, WI USA; 2 Project Ujima, Children’s Wisconsin, Milwaukee, WI USA; 3 Comprehensive Injury Center, Medical College of Wisconsin, Milwaukee, WI USA

**“Nothing in life is to be feared, it is only to be understood. Now is the time to understand more, so that we may fear less.”- Marie Curie.**

For the first time in this readership’s history, we can all claim a shared global experience- COVID-19. Since early 2020, the world has been in the clutches of a pandemic that has claimed many lives, crippled economies, and brought health care systems to their knees. For many of us, the challenges have been daunting as health care priorities have shifted and many are forced to re-imagine what the future of health care and injury prevention will look like.

The pandemic has both impacted the epidemiology of childhood injury and has uncovered health care disparities in childhood injury. Despite stay-at-home orders and many policies limiting how frequently youth were encouraged to be active or gather (school closures, sport cancellations), unintentional injury mortality as well as intentional injury mortality (particularly through gun violence) have increased during the pandemic.(Sutherland et al., 2021; Ahmad & Anderson, 2021) For 26 years, the Injury Free Coalition for Kids® has been among one of the most effective injury prevention programs in the country. Our 30 member hospitals have continued research, education, and advocacy efforts despite the pandemic with one goal in mind: to prevent injury to children.

In this issue, the Injury Free Coalition for Kids® annual meeting supplement in Injury Epidemiology, the reader will get a glimpse of the continuing injury prevention efforts occurring nationally. Given the concurrent gun violence and suicide epidemics in the United States, studies evaluate community violence intervention priorities, examine effective implementation of referrals from a busy Emergency Department to a violence intervention program, and examine one state’s perspective on firearm safety counseling by pediatricians. One study further explores lethal means restriction counseling for suicide prevention and another delves into the infrequently-studied epidemiology of rural youth firearm injury. This issue further highlights injury prevention activities that continued through the pandemic ranging from a unique study on measuring the softness of surfaces for infant sleep safety to the evaluation of a scald burn prevention program. Injury Free Coalition for Kids® members also contributed knowledge to the motor vehicle crash literature through an evaluation of factors associated with Off-Road Vehicle injuries and factors that impact pedestrian and cyclist fatalities.

The pandemic did many things, but it did not hinder the work of our network. Our member hospitals, clinicians, and injury prevention specialists continue to:

1. Research injury- because we know that only through understanding mechanisms, risk factors, and what interventions work can we begin to plan and evaluate injury prevention strategies.
2. Educate about injury- because it is through translation of best practices that we empower our communities to reduce and prevent injury.
3. Advocate for injury prevention/intervention- because it is through data-driven policies that efforts have the biggest impact on keeping our youth safe.

The studies in this issue are merely a fraction of the work being done in the Injury Free Coalition for Kids®. However, the fact that they were conducted and brought to publication during a global pandemic speaks to the continued importance of youth injury prevention and the tireless and fearless perseverance of our authors. Please enjoy these scholarly articles and help us as we strive to make our youth Injury Free.

## Data Availability

Not applicable.
